# Affect, Disordered Eating Attitudes and Behaviors, and Orthorexia Nervosa Among Women: Mediation Through Intuitive Eating

**DOI:** 10.3390/bs15070967

**Published:** 2025-07-16

**Authors:** Mehri Khoshzad, Christophe Maïano, Alexandre J. S. Morin, Annie Aimé

**Affiliations:** 1 Department of Psychoeducation and Psychology, Université du Québec en Outaouais, Gatineau, QC J8X 3X7, Canada; khom11@uqo.ca; 2 Cyberpsychology Laboratory and Department of Psychoeducation and Psychology, Université du Québec en Outaouais (UQO|Campus de Saint-Jérôme), Saint-Jérôme, QC J7Z 0B7, Canada; annie.aime@uqo.ca; 3 Substantive-Methodological Synergy Research Laboratory, Department of Psychology, Concordia University, Montreal, QC H4B 1R6, Canada; alexandre.morin@concordia.ca; 4 Optentia Research Unit, North-West University, P.O. Box 1174, Vanderbijlpark 1900, South Africa

**Keywords:** affect, body mass index, disordered eating attitudes and behaviors, orthorexia nervosa, intuitive eating

## Abstract

Intuitive eating is an adaptive eating style that could help explain part of the relation between affect and eating behaviors. However, research in this area remains limited. The objectives of this study are twofold. First, we examine the relation between affect, disordered eating attitudes and behaviors (DEABs), and orthorexia nervosa (ON). Second, we investigate the mediating role of intuitive eating regarding these relations. A sample of 197 French-speaking Canadian women, aged from 18 to 69, participated in the study. The results showed that negative affect was related to DEABs and ON, but not positive affect. Statistically significant associations were also found between affect (negative and positive) and the four dimensions of intuitive eating. Additionally, negative relations were found between intuitive eating dimensions and most types of eating behaviors (encompassing DEABs and ON), except for dieting. Finally, our results revealed that the relations between affect (positive and negative) and most types of eating behaviors were indirect (i.e., suggesting mediation) via intuitive eating. Based on these results, it seems that interventions addressing affect regulation and eating behaviors could benefit from including a focus on intuitive eating.

## 1. Introduction

Potentially resulting from modern thinness ideals, the past decade has witnessed an increase in disordered eating attitudes and behaviors (Aparicio-Martinez et al., 2019; Holland & Tiggemann, 2016). Moreover, the recent rise of the healthy and clean eating movement has caused some people to become obsessively focused on choosing high-quality healthy foods (Rainey, 2016). This focus, especially when it becomes obsessive, may lead them to restrict their dietary patterns and display pathological orthorexic eating behaviors, commonly referred to as orthorexia nervosa (ON).

Disordered eating attitudes and behaviors (DEABs) encompass a variety of unhealthy weight-control behaviors, such as purging, severe and unwarranted food restriction, and bingeing among people with a distorted perception of their weight and body shape (Izydorczyk & Sitnik-Warchulska, 2018). Contrary to people with DEABs, those displaying pathological orthorexic eating behaviors are not primarily motivated by a desire to lose weight or to control their weight. Moreover, ON is not considered to be a diagnosable eating disorder and its diagnostic criteria have been largely debated in recent years (Dunn & Bratman, 2016; Cena et al., 2019; Donini et al., 2022). Consequently, pathological orthorexic eating behaviors may be measured differently according to the questionnaire used.

The main goals of orthorexic eating behaviors are healthy eating habits, food purity, careful abstinence from foods deemed unhealthy or impure, and appropriate nutritional practices (Costa et al., 2017; Koven & Abry, 2015; Rangel et al., 2012). People with pathological orthorexic eating behaviors are usually more concerned about the quality of their food than with its quantity and they display higher levels of rigidity, such as avoiding going to restaurants and strictly eating what they believe they should eat, etc. (Aksoydan & Camci, 2009; Costa et al., 2017). Although anchored in a desire to be healthy, this intense focus on food quality can lead to obsessive eating behaviors that negatively impact a person’s quality of life (Donini et al., 2004) and health (Selvey & Carey, 2013). For example, Bosi et al. (2007) showed that, due to the elimination of certain food groups, people displaying pathological orthorexic eating behaviors often experience weight loss and malnutrition.

### 1.1. Affect, DEABs, and ON Behaviors

Affect, as the external manifestation of one’s emotions, plays an important role in a person’s psychological, social, and physical life (Fox, 2015). Positive affect (PA) encompasses positive mood states, such as enthusiasm, excitement, desire, interest, happiness, meaningfulness, and confidence (King et al., 2006; Kok et al., 2013; Ramsey & Gentzler, 2015). Conversely, negative affect (NA) encompasses negative mood states, such as distress, depression, anxiety, anger, guilt, fear, and sadness (Bradley, 2003). Many studies have shown that PA can be beneficial for one’s physical and psychological health (Chang et al., 2024; Cohen & Pressman, 2006; Craske et al., 2024; Dockray & Steptoe, 2010; Smith et al., 2024), whereas NA is related to internalizing symptoms (i.e., depression and anxiety) and DEABs (Heron et al., 2014; Palop-Larrea, 2024; Whiteside et al., 2007).

People with DEABs may consume food even when they are not hungry, limit their food intake, or obsess over their diet, and often turn to food to manage their NA (Aytas & Yaprak, 2023; J. Chen et al., 2012). Indeed, in a review of affect regulation models, Spoor et al. (2007) noted that maladaptive eating behaviors are likely to act as a mechanism to relieve NA. In other words, when people with DEABs experience NA, they are likely to turn to overeating, particularly high-calorie and comfort foods, to cope with their NA (Brytek-Matera, 2021). Importantly, eating “forbidden” foods to comfort oneself can itself be associated with post-consumption guilt and negative emotions in some people (Wansink et al., 2003). Koushiou (2016) showed that a high sensitivity to NA can lead people with DEABs to seek immediate relief when experiencing NA. This rapid response to NA may, in turn, trigger impulsive and unhealthy eating behaviors (Koushiou, 2016). Interestingly, none of these associations seem to hold for PA (e.g., Cardoso et al., 2020).

NA is also related to maladaptive strategies among people engaging in ON behaviors (Asarkaya & Arcan, 2021; Barrada & Roncero, 2018; Chace & Kluck, 2022). To manage their NA, people with ON can resort to behaviors ranging from eating healthy foods to engaging in more extreme dieting practices. When people with ON eat unhealthy foods, they are likely to feel guilty and may even punish themselves by further restricting their food intake (Donini et al., 2022; Mathieu, 2005). Eating unhealthy foods may exacerbate their anger, anxiety, stress, and NA (Bratman & Knight, 2004). Moreover, people engaging in ON behaviors could have a reduced capacity to tolerate distress and may engage in maladaptive eating behaviors as a short-term solution to cope with distress, even if these behaviors can have detrimental long-term effects (Hayatbini, 2024; Rand-Giovannetti et al., 2022). The recent results on PA and ON are mixed: Some studies report a negative association between PA and ON behaviors (e.g., Barthels et al., 2019), whereas others reveal a lack of association (e.g., Asarkaya & Arcan, 2021).

### 1.2. Intuitive Eating as a Mediator of the Relations Between Affect and DEABs or ON

Prior research has highlighted an association between adaptive affect regulation and intuitive eating (Howell, 2018; Shateri et al., 2018). Affect regulation helps people cope with stressors and challenges and, thus, represents an essential mechanism underpinning the ability to maintain adequate levels of psychological well-being (Gratz & Roemer, 2004; Werner & Gross, 2010). Several theories suggest that people who lack affective regulation skills can resort to maladaptive eating strategies when faced with NA (Brytek-Matera, 2021; Fairburn et al., 2003; Schmidt & Treasure, 2006), and, more generally, present a higher risk of experiencing DEABs (Arslan et al., 2022; Haedt-Matt & Keel, 2011; Ruscitti et al., 2016; Shouse & Nilsson, 2011). Likewise, research has also shown that affect dysregulation shares a positive association with ON behaviors (Gerges et al., 2023; Obeid et al., 2021). Additionally, individuals with ON seem to be more influenced by affective cues than by their physical hunger and satiety signals (Demirgül & Rigó, 2023).

Intuitive eating represents an adaptive and flexible eating approach that plays a crucial role in eating behaviors by enhancing affect regulation and helping people manage their responses to both PA and NA (Shateri et al., 2018). Intuitive eating has been associated with the ability to accept both negative and positive emotions as inherent aspects of life, which in turn is related to a more adaptive affective response (Shouse & Nilsson, 2011). This eating style reduces reactivity and impulsivity, fostering more balanced responses to NA, which, in turn, can prevent impulsive behaviors like restricted eating or overeating based on affect (Emiroğlu & Aktaç, 2023; Cardoso et al., 2020). Individuals with a propensity for ON behaviors are also considerably less likely to eat intuitively. In fact, ON behaviors prevent them from eating freely or enjoying foods that seem to be less healthy (Rodgers et al., 2021). This contrasts with the restrictive and unhealthy emphasis ON behaviors put on food purity and the avoidance of perceived “unhealthy” foods (Coimbra & Ferreira, 2021; Yakın et al., 2021). By enabling individuals to respond to internal cues rather than external signals and to better differentiate between physical and emotional hunger, intuitive eating should naturally lead to lower DEABs (Hawks et al., 2008) and ON (Anastasiades & Argyrides, 2023; Demirgül & Rigó, 2023).

In sum, research exploring the relations between affect and DEABs or ON behaviors highlights the potential role of intuitive eating as a mediator of the associations between affect, DEAB, and ON. To our knowledge, a single study (Cardoso et al., 2020) has examined intuitive eating as a mediator of the association between affect and DEABs among a sample of 273 women, aged from 18 to 45 years. Their results revealed a positive relation between NA and DEABs and a positive relation between PA and DEABs. Additionally, they revealed a negative association between intuitive eating and DEABs in a way that is consistent with the mediating role of intuitive eating regarding the relation between NA or PA and DEABs. Despite the interest in this study, multiple limitations remain regarding our understanding of the links between affect, intuitive eating, DEABs, and ON. Indeed, no available research has taken into consideration whether and how the results can differ across intuitive eating dimensions (e.g., Swami et al., 2022): (a) unconditional permission to eat (i.e., a person’s willingness to eat when hungry and to refuse to categorize some foods as forbidden); (b) eating for physical rather than emotional reasons (i.e., eating when physically hungry rather than to deal with emotional distress); (c) a reliance on hunger and satiety cues (i.e., a person’s confidence in their internal hunger and satiety cues and their trust in these cues to guide their eating behaviors); and (d) body–food choice congruence (i.e., a person’s tendency to choose foods that honor his/her health and body functioning). Likewise, no available research has considered whether and how associations differ across DEAB dimensions (e.g., Garner et al., 1982): (a) dieting (i.e., the avoidance of fattening foods and concerns about thinness), (b) bulimia and food preoccupation (i.e., thoughts about food related to bulimic behaviors), and (c) oral control (i.e., self-control of eating and perceived pressure from others to gain weight). This lack of consideration of the multidimensional nature of both constructs is concerning, as the dimensions of intuitive eating reflect qualitatively distinct adaptive, flexible, and healthy approaches to eating (Swami et al., 2022), while DEAB dimensions reflect different maladaptive eating patterns, with their own predictors and outcomes (e.g., Stice, 2002; Tylka & Kroon Van Diest, 2015). Therefore, it is unrealistic to expect all associations to generalize across these dimensions. Finally, whether intuitive eating could mediate the relation between affect and ON behaviors also remains unknown.

### 1.3. The Present Study

This study sought to address two objectives. First, we wanted to replicate and extend previous research on the relation between affect and two types of eating behaviors (ON and DEABs), while considering the multidimensional nature of DEABs (Cardoso et al., 2020; J. Chen et al., 2012; Koushiou, 2016). Based on previous results, we hypothesize that: (a) PA will be negatively related to DEABs and ON behaviors (Barthels et al., 2019; Cardoso et al., 2020); and (b) NA will be positively related to DEABs and ON behaviors (Asarkaya & Arcan, 2021; Barrada & Roncero, 2018; Chace & Kluck, 2022; J. Chen et al., 2012; Strahler et al., 2022). Lacking prior empirical and theoretical guidance, we leave as an open research question whether and how these associations differ across different types of DEABs.

As a second objective, we wanted to assess the possible mediator role of intuitive eating in the relation between affect and eating behaviors (i.e., DEABs and ON behaviors), while considering the multidimensional nature of DEABs and intuitive eating. Based on Cardoso et al.’s (2020) study, we hypothesize that intuitive eating will mediate the relations between PA and NA on the one hand, and DEABs and ON behaviors on the other hand. Lacking prior empirical and theoretical guidance, we leave as an open research question whether and how this mediator role differs or generalizes across the different dimensions of intuitive eating and DEABs.

**Figure 1:**
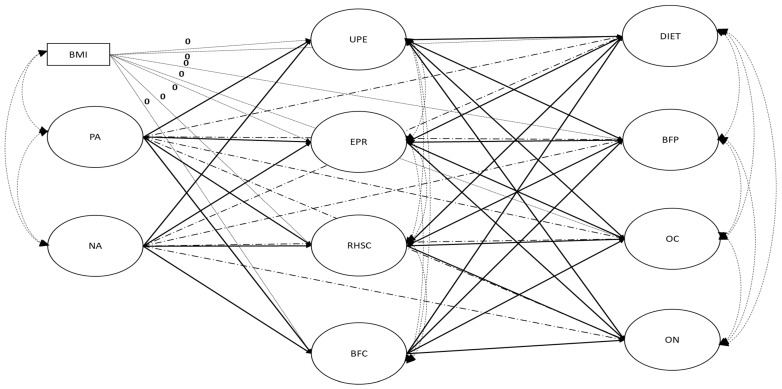
Illustration of the hypothesized fully and partially mediated models, including body mass index as a control variable. Notes: PA = positive affect; NA = negative affect; UPE = unconditional permission to eat; EPR = eating for physical rather than emotional reasons; RHSC = reliance on hunger and satiety cues; BFC = body–food choice congruence; DIET = dieting; BFP = bulimia food preoccupation; OC = oral control; ON = orthorexia nervosa. The full arrows reflect a fully mediated model. Partial mediation will be tested by including the paths depicted by the dashed arrows. Finally, the association between body mass index and the mediators and outcomes will be tested by contrasting the models, including comparing the dotted arrows with models wherein these paths are constrained to zero.

Figure 1 illustrates the *a priori* model that underpins this study. In this figure, the full arrows represent a fully mediated (FM) model. This model suggests that: (a) higher levels of NA will be related to lower levels of intuitive eating, and that higher levels of intuitive eating will be related to higher levels of DEABs and ON behaviors; and (b) higher levels of PA will be related to higher levels of intuitive eating, which will, in turn, be related to lower levels of DEABs and ON behaviors. An alternative model of partial mediation (PM) will also be examined by incorporating the dashed arrows included in Figure 1 to depict direct relations between affect, DEABs, and ON behaviors. Finally, to assess and account for the possible confounding effect of body mass index (BMI) on the observed associations, we will investigate the relevance of controlling for this variable (see the dotted lines in Figure 1). Past research suggests that individuals with a higher BMI tend to display lower levels of intuitive eating (Van Dyke & Drinkwater, 2014; Denny et al., 2013) and higher levels of DEABs (Goldschmidt et al., 2008; Al Banna et al., 2021) and ON behaviors (Brytek-Matera et al., 2020; Bundros et al., 2016). We will, thus, contrast a model in which the effects of BMI are freely estimated with one in which these effects are constrained to zero to assess the relevance of retaining this control in the model.

## 2. Method

### 2.1. Participants and Procedure

The participants were 197 French-speaking women, recruited in the Canadian province of Quebec, aged between 18 and 69 years old (M*_age_* = 33.30, SD*_age_* = 11.37), with a BMI (estimated based on their self-reported height and weight) ranging from 15.24 to 55.82 kg/m^2^ (M*_BMI_* = 24.72, SD*_BMI_* = 6.43). This study was approved by the research ethics committee at the university of three of the authors (#2019-156, 3090; #2025-4007). This convenience sample was recruited at the authors’ university, from within the community, and from private clinics. The participants were invited by using generic announcements posted in a local newspaper and disseminated via emails, social networks, and websites by community organizations and private clinics. The participants had to be at least 18 years old and complete an online informed consent form. Then, they anonymously completed online questionnaires, administered using LimeSurvey.

### 2.2. Measures

#### 2.2.1. DEABs

The French version (Leichner et al., 1994) of the Eating Attitudes Test-26 (EAT-26; Garner et al., 1982) was used. This questionnaire comprises 26 items measuring the following DEABs: (a) dieting (DIET; 13 items; “I engage in dieting behavior”); (b) bulimia food preoccupation (BFP; 6 items; “I have gone on eating binges where I feel that I may not be able to stop”); and (c) oral control (OC; 7 items; “I display self-control around food”). The items were scored on a six-point scale (i.e., 1 = *never* = 1 to 6 = *always*). The composite reliability (McDonald, 1970) of the three scales is acceptable to excellent (DIET: ω = 0.939; BFP: ω = 0.946; OC: ω = 0.785).

#### 2.2.2. Intuitive Eating

The French version (Carbonneau et al., 2016) of the Intuitive Eating Scale-2 (IES-2; Tylka & Kroon Van Diest, 2013) was used. This questionnaire comprises 23 items measuring: (a) unconditional permission to eat (UPE; 6 items; “I allow myself to eat what food I desire at the moment”); (b) eating for physical rather than emotional reasons (EPR; 8 items; “I find myself eating when I am stressed out, even when I’m not physically hungry”); (c) reliance on hunger and satiety cues (RHSC; 6 items; “I trust my body to tell me when to eat”); and (d) body–food choice congruence (BFC; 3 items; “Most of the time, I desire to eat nutritious foods”). The items were scored on a 5-point scale (i.e., 1 = *strongly disagree* to 5 = *strongly agree*). The composite reliability (McDonald, 1970) of the four scales is excellent (UPE: ω = 0.891; EPR: ω = 0.962; RHSC: ω = 0.956; BFC: ω = 0.933).

#### 2.2.3. ON Behaviors

The orthorexia nervosa (8 items; “I feel guilty when I eat food that I do not consider healthy”) scale of the French-speaking Canadian version (Maïano et al., 2022) of the Teruel Orthorexia Scale (TOS; Barrada & Roncero, 2018) was used. The items were scored on a 4-point scale (0 = *completely disagree* to 3 = *completely agree*). The composite reliability (McDonald, 1970) of this scale is excellent (ω = 0.936).

#### 2.2.4. Positive and Negative Affect

The French version (Gaudreau et al., 2006) of the positive and negative affects scale (PANAS; Watson et al., 1988) was used. This questionnaire comprises 20 items measuring PA (10 items; “Interested”, “proud”, etc.) and NA (10 items; “Irritable”, “Ashamed”, etc.). The items were scored on a 5-point scale (1 = *not at all or very slightly* to 5 = *extremely*). The composite reliability (McDonald, 1970) of the two scales is excellent (PA: ω = 0.920; NA: ω = 0.939).

### 2.3. Analyses

All the analyses were realized in Mplus 8.11 (Muthén & Muthén, 2024), using the robust weighted least squares estimator, with mean and variance adjusted statistics (WLSMV). The model was estimated using all information present at the item level (0–1.02%, M = 0.53) and the few missing responses were handled using the missing data algorithm implemented in Mplus, with WLSMV estimation (Asparouhov & Muthén, 2010).

#### 2.3.1. Preliminary Analyses

The preliminary confirmatory factor analysis (CFA) was estimated to confirm the validity and reliability of the ten latent variables (i.e., PA, NA, UPE, EPR, RHSC, BFC, DIET, BFP, OC, ON) used in our main analyses. In regard to this CFA model, the responses to the: (a) IES-2 were explained by four correlated latent factors (UPE, EPR, RHSC, BFC), defined only by their a priori items; (b) EAT-26 were explained by three correlated latent factors (DIET, BFC, and OC), defined only by their a priori items; (c) PANAS were explained by two correlated latent factors (PA and NA), defined only by their a priori items; and (d) ON was explained by one latent factor, defined only by its a priori items. The composite reliability of the latent variables was calculated using McDonald’s (1970) omega (ω) coefficient. As recommended (e.g., Hu & Bentler, 1999; Marsh et al., 2005), the model fit was assessed using the comparative fit index (CFI), the Tucker–Lewis index (TLI), and the root mean square error of approximation (RMSEA). Values ≥0.90 or >0.95 for the CFI and TLI and ≤0.08 or <0.06 for the RMSEA indicated an acceptable and excellent fit, respectively.

#### 2.3.2. FM and PM Models

Our main analysis relied on structural equation modeling (SEM), which was used in contrast to our a priori FM and PM models (see Figure 1). First, these models were estimated without controlling for BMI. To enable a comparison between the models to be conducted, we still included BMI in these models, allowing it to correlate freely with the exogenous predictors (PA and NA), but constrained its associations with the endogenous (mediators and outcomes) factors to zero. However, their regression paths to the mediators and the outcomes were constrained to zero. Only the best of these two models (FM or PM) was retained for the second step. In this second step, the retained FM or PM model was contrasted with an alternative model in which the associations between BMI and the endogenous (mediators and outcomes) factors were freely estimated. In our main analyses, the model fit was assessed as in our preliminary analyses, and the model comparisons relied on an examination of the changes (∆) in the CFI, TLI, and RMSEA. Changes were considered relevant when ∆CFI and ∆TLI were >0.01, and/or ∆RMSEA was >0.015 (F. F. Chen, 2007; Cheung & Rensvold, 2002). Finally, the statistical significance of suspected indirect relations (i.e., when the direct paths linking the predictor and mediator, and the mediator and outcome, are both statistically significant) was estimated using bias-corrected (BC) bootstrap 95% confidence intervals (95% CI), based on 1,000 bootstrap samples (Cheung & Lau, 2008; Lau & Cheung, 2012). An indirect relation is considered statistically significant when its CI excludes zero.

## 3. Results

### 3.1. Factor Validity and Reliability of the Latent Variables

The CFA model resulted in a satisfactory level of fit to the data (χ^2^ = 4231.031, df = 2804, *p* < 0.001; CFI = 0.930, TLI = 0.927, RMSEA = 0.051; RMSEA 90% CI = 0.048–0.054). As shown in Table S1 of the Supplementary Materials, the results revealed well-defined (λ = 0.248 to 0.979, M_λ_ = 0.772) and reliable (ω = 0.785 to 0.962, M_ω_ = 0.921) latent factors.

### 3.2. Variable Correlations

The correlations are reported in Table 1. First, the results show that DIET, BFP, and OC are significantly correlated with each other and with most other variables. Second, the results show that ON is significantly correlated with most of the variables, except for PA and BMI. Third, the results show that UPE, EPR, RHSC, and BFC are significantly correlated with each other and with most of the other variables. Finally, PA and NA are significantly correlated, NA is significantly correlated with the other variables except for BFC, and PA is only significantly correlated with RHSC and BFC.

### 3.3. Comparison of the Fully and Partially Mediated Models

The FM^1^ model had a satisfactory fit to the data (χ^2^ = 4382.617, df = 2887, *p* < 0.001; CFI = 0.927, TLI = 0.924, RMSEA = 0.051; RMSEA 90% CI = 0.048–0.054), and adding direct paths between the predictors and outcomes (PM^2^: χ^2^ = 4386.363, df = 2879, *p* < 0.001; CFI = 0.926, TLI = 0.923, RMSEA = 0.052; RMSEA 90% CI = 0.048–0.055) did not result in an improved level of fit (ΔCFI = −0.001, ΔTLI = −0.001, ΔRMSEA = +0.004). Therefore, the most parsimonious FM solution was retained for further analyses. Freely estimating the relations between BMI and the endogenous factors in the FM^3^ model (χ^2^ = 4357.591, df = 2879, *p* < 0.001; CFI = 0.927, TLI = 0.924, RMSEA = 0.051; RMSEA 90% CI = 0.048–0.054) also did not result in an improved level of fit to the data (ΔCFI = 0.000, ΔTLI = 0.000, ΔRMSEA = 0.000). The FM model that did not control for BMI was, thus, retained as our final model.

**Figure 2:**
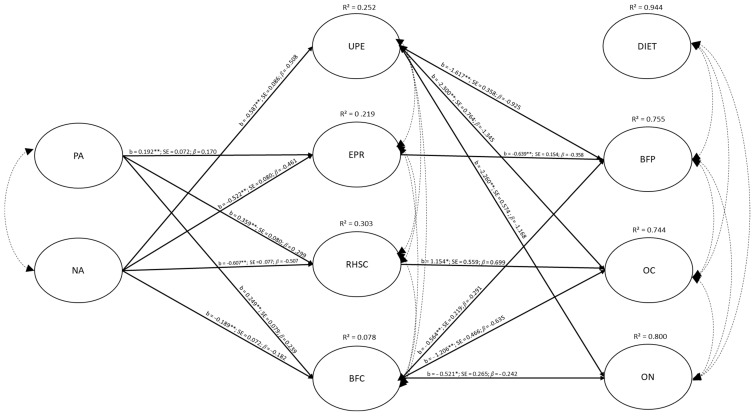
Unstandardized and standardized parameter estimates in the fully mediated model, excluding body mass index as a control variable. Notes: * *p* ≤ 0.05; ** *p* ≤ 0.01; b = unstandardized parameter; SE = standard error; PA = positive affect; NA = negative affect; UPE = unconditional permission to eat; EPR = eating for physical rather than emotional reasons; RHSC = reliance on hunger and satiety cues; BFC = body–food choice congruence; DIET = dieting; BFP = bulimia food preoccupation; OC = oral control; ON = orthorexia nervosa. Only statistically significant paths are presented in the figure. Covariances between latent variables are not presented, but are available upon request from the corresponding author.

The direct and indirect relations between the variables in the FM model that did not control for BMI are presented in Table 2. In addition, a visual representation of the direct and significant relations in this model is presented in Figure 2. This model accounted for 8% to 30% of the variance in the participants’ intuitive eating factors, ranging from a low 7.8% for BFC to a moderate 21.9% for EPR, 25.2% for UPE, and 30.3% for RHSC. The model also explained 74.4% of the variance in OC, 75.5% of the variance in BFP, and 80.0% of the variance in ON. Surprisingly, the model also explained 94.4% of the variance in the participants’ DIET, even though none of the predictions involving this factor were statistically significant. This last result suggests that the lack of association with DIET may reflect the low prevalence of severe dieting in the convenience sample.

More precisely, our results show that: (a) PA significantly and positively predicted EPR, RHSC, and BFC; (b) NA significantly and negatively predicted all intuitive eating factors; (c) UPE, EPR, and BFC significantly and negatively predicted BFP; (d) RHSC significantly and positively predicted OC; and (e) UPE and BFC significantly and negatively predicted OC and ON. Based on these direct effects, the relevance of potential indirect effects was calculated. These results, reported in Table 2, revealed a significant positive indirect association in terms of: (a) UPE on the relation between NA and BFP; (b) UPE on the relation between NA and OC; (c) UPE on the relation between NA and ON; (d) EPR on the relation between NA and BFP; (e) BFC on the relation between NA and OC; (f) BFC on the relation between NA and BFP; (g) BFC on the relation between NA and ON; and (h) RHSC on the relation between PA and OC. Finally, our results also revealed a significant negative indirect association in terms of: (a) EPR on the relation between PA and BFP; (b) BFC on the relation between PA and BFP; (c) BFC on the relation between PA and OC; (d) BFC on the relation between PA and ON; and (e) RHSC on the relation between NA and OC.

## 4. Discussion

### 4.1. Relations Between Affect and DEABs or ON Behaviors

The first objective of this study was to examine the relation between affect, DEABs (i.e., DIET, BFP, OC), and ON. The correlational analyses revealed that NA was significantly related to all types of DEABs, but not PA. These positive associations between NA and DEABs are consistent with prior research, suggesting that women with higher levels of NA tend to display higher levels of DEABs (Cardoso et al., 2020; Cooper et al., 2014). However, the lack of associations involving PA is not consistent with Cardoso et al.’s (2020) finding that women with higher levels of PA tend to display lower levels of DEABs. This discrepancy may be attributed to differences in the way DEABs were measured in both studies. Indeed, our study differentiated among three types of DEABs, whereas Cardoso et al. (2020) relied on a less precise aggregated score of disordered eating pathology. Additionally, our sample is older (33.30 years) than Cardoso et al.’s (2020; 24.10 years) and, as reported in Pinquart’s (2001) meta-analysis, older participants tend to report higher levels of NA and lower levels of PA. In sum, the present findings suggest that NA is related to all types of DEABs and is more likely than PA to lead to disordered eating (Bradley, 2003).

Second, in line with previous studies (Awad et al., 2021; Wee, 2023), the correlational analyses revealed a positive relation between NA and ON behaviors. Vuillier et al. (2020) suggested that people can resort to strict healthy eating behaviors as a coping mechanism for NA, even if these behaviors may be detrimental to their well-being. This rigid adherence to a healthy diet can foster a sense of competency or even superiority, which not only temporarily alleviates NA, but also enhances PA, ultimately reinforcing a harmful cycle (Donini et al., 2004; Hayatbini, 2024). Additionally, in line with a recent study (Asarkaya & Arcan, 2021), no relation was found between PA and ON.

### 4.2. The Mediating Role of Intuitive Eating in the Relations Between Affect and DEABs or ON Behaviors

The second objective of this study was to investigate the indirect role of intuitive eating regarding the association between affect and eating behaviors (DEABs and ON). Our results showed that affect was significantly associated with all the dimensions of intuitive eating. More precisely, PA was positively associated with EPR, RHSC, and BFC (leaving out only UPE), while NA was negatively related to all the dimensions of intuitive eating. As suggested by Cardoso et al. (2020), these results could imply that women with NA may be less responsive to their body cues and exhibit less intuitive eating, while those with PA may be more attuned to their physical body cues and have a greater tendency to engage in intuitive eating. Moreover, most dimensions of intuitive eating were negatively related to the BFP and OC types of DEABs, as well as to ON, with only a few exceptions. For instance, whereas UPE and BFC were negatively related to BFP, OC, and ON, EPR was only negatively related to BFP, and RHSC was only positively related to OC. Globally, the relations found between intuitive eating dimensions, ON, and the BFP and OC types of DEABs emphasize that both adaptive and maladaptive eating practices could lay at the interface of food-related attention (intuitive eating) and control (ON, BFP, and OC). One may see these variables as reflecting distinct “eating styles,” with intuitive eating defined by internal regulation and emotional balance, whereas types of DEABs and ON entail more control and anxiety related to food. However, both were related, suggesting that they may be part of an underlying eating regulation mechanism, driven in part by affect.

None of the dimensions of intuitive eating were related to dieting. While this result could suggest that clinically extreme dieting behaviors like fasting are less likely to be observed in normative populations (Brown et al., 2015; Hill, 2002), it could also indicate that intuitive eating and dieting are two different eating styles that need to be assessed separately. Importantly, normative forms of dieting are accepted and even encouraged in some cultures and religions (Brownell, 1991; Palmer, 2017). In these contexts, dieting may lead to positive attention and social reinforcement, thus not being influenced by internal factors, such as intuitive eating. Furthermore, intuitive eating is widely acknowledged as a counterpoint to traditional dieting. It advocates a balanced and varied diet, based on personal taste preferences and natural hunger and fullness signals rather than imposing rigid calorie limitations, set portion sizes, or eliminating food categories. This approach emphasizes flexibility and trust in one’s own body (Tribole & Resch, 2020), which may simply be unrelated (neither positively nor negatively) to more extreme forms of dieting.

Our results also showed that intuitive eating could act as a mediator in the relation between affect and DEABs/ON. The identification of intuitive eating as a mediator of these associations helps improve our understanding of the mechanisms likely to be involved in the role played by affect in the emergence of maladaptive eating behaviors. To the best of our knowledge, the present study is the first to examine the mediating role of the various dimensions of intuitive eating regarding these associations. According to most affect regulation models (Spoor et al., 2007), intuitive eating could encourage people to be less reactive to their affects and more attuned to the needs of their body, which, in turn, could help limit DEABs. Our findings provide some support to these theoretical propositions by highlighting a positive indirect role of UPE, EPR, and BFC in the relations between affect, BFP, OC, and ON. Indeed, previous research has also shown that intuitive eating is related to higher levels of body acceptance and trust in internal cues and less related to external pressures and affective cues (Caferoglu & Toklu, 2022; Cardoso et al., 2020; Linardon et al., 2021; Yılmaz & Arpa Zemzemoglu, 2021). Similarly, Atalay et al. (2020) showed that intuitive eating is related to higher levels of body trust and attention toward individual’s needs and lesser related to seeking social approval or striving for an ideal body. Finally, this positive indirect role of intuitive eating dimensions could also be temperamental and could be explained by a higher ability of women to regulate their eating behavior.

Our results showed that UPE, a key subcomponent of intuitive eating, seems to play a strong positive indirect role in the relations between NA and ON behaviors. This finding aligns with past research (Demirgül & Rigó, 2023; Rodgers et al., 2021) showing that ON behaviors share a strong negative relation with UPE. This finding supports the notion that individuals with ON could struggle with allowing themselves to eat foods they judge as forbidden. Not only will they tend to choose healthy foods, but they will also avoid eating freely, often influenced by guilt associated with certain foods (Thorne et al., 2022). When people do not allow themselves to eat freely and unconditionally, which is a core element of intuitive eating, this may contribute to the development of ON behaviors (Anastasiades & Argyrides, 2022; Rodgers et al., 2021). This suggests that the inability to engage in intuitive eating, which includes giving oneself unconditional permission to eat, may reinforce ON behaviors.

Finally, our results showed that UPE and BFC were negatively related to most DEABs and ON behaviors, whereas EPR and RHSC were only related to BFP (negatively) and OC (positively), respectively. This may be explained by the fact that UPE and BFC refers to general and internal eating styles (i.e., eating when hungry, a lack of forbidden foods, and choosing foods honoring health and body functioning), in contrast to BFP, OC, and ON behaviors, which refer to externally regulated eating behaviors. Conversely, EPR and RHSC refer to physical eating cues directly related to BFP and OC, which refer to thoughts about food and eating self-control.

### 4.3. Limitations of the Present Study

Although this study offers valuable scientific insights, its findings should be interpreted with caution due to its limitations. One of those limitations is our reliance on a relatively small convenience sample of French-speaking Canadian women. Therefore, it remains unknown whether our results would generalize to younger or older populations and people from other countries and cultures (such as Asian, African populations, etc.). Importantly, our sole focus on women also significantly impacts the generalizability of the findings, making it impossible to assume that they would, or would not, generalize to men and nonbinary individuals. Considering that social pressure to adhere to a diet and attain an ideal body shape tends to be lower among men than women (Gast et al., 2012), men tend to rely on higher levels of intuitive eating (Tylka & Kroon Van Diest, 2013) and to report lower levels of ON (Sanlier et al., 2016). These differences clearly highlight that replication efforts involving samples of men, women, and nonbinary individuals are required to achieve a more comprehensive understanding of the situation. Lastly, our relatively small sample might have interfered with our ability to identify additional statistically significant (albeit smaller) effects and to test more comprehensive models. It will, thus, be important for future studies to rely on larger, more diversified, and potentially more representative samples.

Another limitation comes from the cross-sectional nature of our study, which precludes the precise determination of causal directions, or even simpler tests of directionality, regarding the examined relations. Without longitudinal data, it is impossible to assess the directionality of the relations (e.g., could ON predict intuitive eating as well?) or to establish causality (which requires experimental data). For instance, Wee (2023) suggested that there might be a reciprocal relation between emotions and ON behaviors, which requires further investigation. Moreover, other mediating factors, such as self-esteem, body image, self-regulation, and emotional regulation, could also be considered as potential mediators alongside intuitive eating and should be considered in future research.

Finally, given that ON behaviors may be measured differently according to the questionnaire used, the present results are specific to the TOS and should not be generalized to other measures of ON, at least prior to their replication with other measures.

## 5. Conclusions

Maladaptive eating behaviors have long been associated with affect regulation and are considered to represent a way to relieve NA (Spoor et al., 2007). In this study, women appeared particularly at risk of resorting to binge eating or to behaviors characteristics of ON to cope with NA. The mediator role of intuitive eating found in this study suggests that the relation between affect and maladaptive eating is complex and can be influenced by various types of eating strategies among women. Along with intuitive eating, some other protective factors like self-esteem, positive body image, and emotional regulation could play a protective role in this relation. Moreover, fostering intuitive eating in adults could represent an effective way to prevent maladaptive eating and even eating disorders. Along that line, the “My Body Knows When” program, created for military spouses, has demonstrated promising outcomes in enabling adult women to reject dieting attitudes and adopt better eating habits (Cole & Horacek, 2010). Intuitive eating appears to be a promising protective factor which, together with positive body image dimensions, such as body functionality and body appreciation, could help women stand against the environmental pressure they face to control their eating and their weight (Linardon, 2022; Linardon & Mitchell, 2017).

## Figures and Tables

**Table 1:** Correlations between latent and observed variables.

	DIET	BFP	OC	ON	UPE	EPR	RHSC	BFC	PA	NA	BMI
**DIET**	-										
**BFP**	0.800	-									
**OC**	0.711	0.707	-								
**ON**	0.779	0.818	0.685	-							
**UPE**	−0.842	−0.736	−0.678	−0.823	-						
**EPR**	−0.384	−0.492	−*0.097*	−0.212	0.185	-					
**RHSC**	−0.648	−0.644	−0.386	−0.512	0.593	0.507	-				
**BFC**	−*0.008*	−*0.083*	−*0.090*	0.162	−0.222	0.315	0.251	-			
**PA**	*0.007*	−*0.068*	*0.051*	−*0.003*	*0.048*	*0.102*	0.208	0.242	-		
**NA**	0.603	0.550	0.462	0.514	−0.442	−0.427	−0.467	−*0.130*	0.143	-	
**BMI** **(obs)**	0.206	*0.096*	−0.279	*0.080*	−*0.042*	−0.219	−0.201	−*0.115*	−*0.026*	0.212	-

Notes: DIET = dieting; BFP = bulimia food preoccupation; OC = oral control; ON = orthorexia nervosa; UPE = unconditional permission to eat; EPR = eating for physical rather than emotional reasons; RHSC = reliance on hunger and satiety cues; BFC = body–food choice congruence; PA = positive affect; NA = negative affect; BMI = body mass index; obs = observed variable. Underlined and italicized correlations are non-significant.

**Table 2:** Direct and indirect relations between the variables in the fully mediated model, excluding body mass index as a control variable.

**Direct Relations**	**b**	**SE**	**p**	**β**	**Direct Relations**	**b**	**SE**	**p**	**β**
PA → UPE	0.085	0.076	0.264	0.073	EPR → DIET	−1.027	0.684	0.133	−0.275
PA → EPR	0.192	0.072	0.007	0.170	EPR → BFP	−0.639	0.154	0.000	−0.358
PA → RHSC	0.359	0.080	<0.001	0.299	EPR → OC	−0.064	0.164	0.697	−0.037
PA → BFC	0.249	0.079	0.002	0.239	EPR → ON	−0.287	0.184	0.120	−0.145
NA → UPE	−0.587	0.086	<0.001	−0.508	RHSC → DIET	1.481	1.250	0.236	0.420
NA → EPR	−0.522	0.080	<0.001	−0.461	RHSC → BFP	0.375	0.265	0.158	0.222
NA → RHSC	−0.607	0.077	<0.001	−0.507	RHSC → OC	1.154	0.559	0.039	0.699
NA → BFC	−0.189	0.072	0.009	−0.182	RHSC → ON	0.736	0.386	0.057	0.394
UPE → DIET	−4.499	2.651	0.090	−1.233	BFC → DIET	−1.582	1.080	0.143	−0.390
UPE → BFP	−1.617	0.358	0.000	−0.925	BFC → BFP	−0.564	0.219	0.010	−0.291
UPE → OC	−2.300	0.764	0.003	−1.345	BFC → OC	−1.206	0.466	0.010	−0.635
UPE → ON	−2.260	0.574	0.000	−1.168	BFC → ON	−0.521	0.265	0.049	−0.242
**Indirect Relations**	**b**	**BCB 95% CI**			**Indirect Relations**	**b**	**BCB 95% CI**		
PA → EPR → BFP	−0.123	−0.435; −0.036			NA → UPE → BFP	0.949	0.793; 1.412		
PA → BFC → BFP	−0.140	−0.396; −0.039			NA → EPR → BFP	0.333	0.183; 0.590		
PA → RHSC → OC	0.414	0.084; 1.336			NA → BFC → BFP	0.107	0.023; 0.438		
PA → BFC → OC	−0.300	−0.846; −0.134			NA → UPE → OC	1.350	0.776; 3.044		
PA → BFC → ON	−0.130	−0.378; −0.033			NA → RHSC → OC	−0.701	−2.055; −0.245		
					NA → BFC → OC	0.228	0.089; 0.485		
					NA → UPE → ON	1.326	0.933; 2.475		
					NA → BFC → ON	0.099	0.005; 0.263		

Notes: b = unstandardized parameter; SE = standard error; β = standardized parameter; PA = positive affect; UPE = unconditional permission to eat; EPR = eating for physical rather than emotional reasons; RHSC = reliance on hunger and satiety cues; BFC = body–food choice congruence; DIET = dieting; BFP = bulimia food preoccupation; OC = oral control; ON = orthorexia nervosa; NA = negative affect; BCB 95% CI = bias-corrected bootstrap 95% confidence interval.

## Data Availability

The data is unavailable due to ethical restrictions.

## Notes

1 A power estimation based on an online calculator developed by Preacher and Coffman (2006) was conducted using α = 0.05, a null RMSEA of 0.08, the sample size, and the model’s *df* and RMSEA value. The results revealed a power of 100% and indicated that a sample of 20 participants would be necessary to reach a power of 80%.

2 The results revealed a power of 100% and indicated that a sample of 20 participants would be necessary to reach a power of 80%.

3 See Note 2.

## Supplementary Materials

The following supporting information can be downloaded at: https://www.mdpi.com/article/10.3390/bs15070967/s1, Table S1: Standardized Parameter Estimates from the Confirmatory Factor Analytic Model of the Ten Latent Variables.
