# Supplementary material for: Affect, Disordered Eating Attitudes and Behaviors, and Orthorexia Nervosa Among Women: Mediation Through Intuitive Eating
Source: Behav Sci (Basel). 2025 Jul 16;15(7):967. doi: 10.3390/bs15070967 (PMC12292590; doi:10.3390/bs15070967)
Supplement: Supplementary file 1 [file behavsci-15-00967-s001.zip › behavsci-3647718-supplementary.pdf]

Online Supplements for:

Affect, Disordered Eating Attitudes and Behaviors and Orthorexia Nervosa Among Women: Mediation Through Intuitive Eating

Table S1

Standardized Parameters Estimates from the Confirmatory Factor Analytic Model of the Ten Latent Variables

Intuitive eating						Disordered Eating Attitudes and Behaviors					Affect				Orthorexia Nervosa		
Items	UPE (λ)	EPR (λ)	RHSC (λ)	BFC (λ)	δ	Items	DIET (λ)	BFP (λ)	OC (λ)	δ	Items	PA (λ)	NA (λ)	δ	Items	λ	δ
1	.632				.600	1	.848			.280	1	.486		.764	1	.763	.418
2	.755				.429	6	.674			.546	3	.649		.579	2	.854	.271
3	.924				.145	7	.702			.507	5	.777		.396	3	.741	.451
4	.799				.362	10	.867			.249	9	.858		.263	4	.798	.363
5	.720				.481	11	.893			.203	10	.874		.237	5	.866	.249
6	.702				.507	12	.795			.368	12	.403		.837	6	.657	.568
7		.925			.145	14	.887			.213	14	.819		.329	7	.951	.096
8		.890			.208	16	.618			.618	16	.871		.242	8	.785	.384
9		.946			.106	17	.522			.728	17	.669		.552			
10		.936			.123	22	.861			.258	19	.823		.323			
11		.746			.444	23	.804			.354	2		.825	.319			
12		.820			.328	24	.684			.532	4		.686	.529			
13		.843			.289	25	.261			.932	6		.927	.141			
14		.860			.261	3		.923		.148	7		.828	.315			
15			.896		.197	4		.780		.392	8		.608	.630			
16			.779		.393	9		.820		.328	11		.718	.484			
17			.949		.100	18		.908		.175	13		.918	.157			
18			.853		.273	21		.932		.131	15		.770	.407			
19			.861		.259	26		.799		.361	18		.662	.562			
20			.960		.078	2			.897	.196	20		.815	.336			
21				.819	.330	5			.408	.834							
22				.979	.041	8			.645	.584							
23				.916	.161	13			.248	.938							
						15			.283	.920							
						19			.654	.572							
						20			.843	.289							
ω	.891	.962	.956	.933			.939	.946	.785			.920	.939			.936	

Notes. λ = factor loadings; UPE = Unconditional Permission to Eat; EPR = Eating for Physical Rather than Emotional Reasons; RHSC = Reliance on Hunger and Satiety Cues; BFC = Body-Food Choice Congruence; δ = Uniquenesses; DIET = Dieting; BFP = Bulimia-Food Preoccupation; OC = Oral Control; PA = positive affect; NA = negative affect; ω = McDonald's omega.
